# Taking a deeper look: Quantifying the differences in fish assemblages between shallow and mesophotic temperate rocky reefs

**DOI:** 10.1371/journal.pone.0206778

**Published:** 2019-03-15

**Authors:** Joel Williams, Alan Jordan, David Harasti, Peter Davies, Tim Ingleton

**Affiliations:** 1 Fisheries Research, NSW Department of Primary Industries, Nelson Bay, New South Wales, Australia; 2 New South Wales Office of Environment and Heritage, New South Wales, Sydney, Australia; Department of Agriculture and Water Resources, AUSTRALIA

## Abstract

The spatial distribution of a species assemblage is often determined by habitat and climate. In the marine environment, depth can become an important factor as declining light and water temperature leads to changes in the biological habitat structure. To date, much of the focus of ecological fish research has been based on reefs in less than 40 m with little research on the ecological role of mesophotic reefs. We deployed baited remote underwater stereo video systems (stereo-BRUVS) on temperate reefs in two depth categories: shallow (20–40 m) and mesophotic (80–120 m), off Port Stephens, Australia. Sites were selected using data collected by swath acoustic sounder to ensure stereo-BRUVS were deployed on reef. The sounder also provided rugosity, slope and relief data for each stereo-BRUVS deployment. Multivariate analysis indicates that there are significant differences in the fish assemblages between shallow and mesophotic reefs, primarily driven by *Ophthalmolepis lineolatus* and *Notolabrus gymnogenis* only occurring on shallow reefs and schooling species of fish that were unique to each depth category: *Atypichthys strigatus* on shallow reefs and *Centroberyx affinis* on mesophotic reefs. While shallow reefs had a greater species richness and abundance of fish when compared to mesophotic reefs, mesophotic reefs hosted the same species richness of fishery-targeted species. *Chrysophrys auratus* and *Nemodactylus douglassii* are two highly targeted species in this region. While *C*. *auratus* was numerically more abundant on shallow reefs, mesophotic reefs provide habitat for larger fish. In comparison, *N*. *douglassii* were evenly distributed across all sites sampled. Generalized linear models revealed that depth and habitat type provided the most parsimonious model for predicting the distribution of *C*. *auratus*, while habitat type alone best predicted the distribution of *N*. *douglassii*. These results demonstrate the importance of mesophotic reefs to fishery-targeted species and therefore have implications for informing the management of these fishery resources on shelf rocky reefs.

## Introduction

The spatial distribution of a species assemblage is strongly determined by habitat and physical conditions [1,2], and in the marine environment depth is an important factor [3–5]. On the inner continental shelf the decreased light conditions with increasing water depth results in a change from macroalgal to sessile invertebrate dominated habitat composition [6,7]. In temperate waters this change occurs at depths of around 20–30 m, although variations occur reflecting localised conditions [8–10]. To date, much of the research on rocky reefs on the inner shelf has been focussed on reefs in depths less than 20 m reflecting the widespread use of scuba to conduct such surveys. There are few standardised tools to quantitatively survey fish at greater depths. This is despite the significant range of pressures on deeper rocky reefs across the continental shelf, such as commercial and recreational fishing that target reef-associated species [11–13]. In recent decades there has been anecdotal evidence that recreational fishers have an increased technical capacity such as side-scan or multibeam sonar and electric reels and are therefore, able to target deeper reefs. Previously these reefs may have provided refuge for older, mature individuals [14,15]. Thus, with increasing recreational fishing activity at these depths it is important that we gain a better understand on the abundance and diversity of fishes at depths >30m.

Mesophotic reefs are those characterised by the presence of light-dependent corals and associated communities often between the depths of 30–40 and 150 m in tropical and subtropical regions of the world [7,16–19]. Furthermore, there is now a broad understanding that this zone can be divided into the upper and lower mesophotic zone with a transition zone at ~60 m depending on water clarity and temperature [19]. The recent worldwide expansion of multibeam acoustic surveys of continental shelf waters has revealed that they form extensive areas of habitat in many regions [9,20,21]. Mesophotic reefs are often continuous with shallow reefs, resulting in potentially strong connectivity across a large depth gradient, a feature common in the Great Barrier Reef, Australia, north eastern Brazil and the Hawaiian Archipelago [6,7,22,23]. They can also form discontinuous areas that are interspersed among areas of unconsolidated habitat, such as in the Gulf of Carpentaria, Australia [17,24]. While the number of studies on mesophotic reef has increased significantly over the past decade [18,19], the majority of this research is focussed in the tropics where such reefs usually contain scleractinian corals [17,18]. Conversely, temperate mesophotic ecosystems tend to be dominated by sponges and octocorals [9,20,25]. In comparison to the tropical mesophotic coral ecosystems, little is known about temperate mesophotic ecosystems, particularly the link between fish assemblages, habitat structure, and connectivity with shallower reefs [13,25].

Temperate mesophotic reefs have important biodiversity, social and economic values [16,25], so understanding the characteristics of their associated fish assemblages is fundamental to effectively managing them. Habitat type (coral, sponge, bare) and complexity (relief, rugosity, curvature) are known to be important in structuring fish assemblages [26–31]. Habitat complexity is considered as the variance in surface structure of the reef and can be defined in terms of relief, slope, rugosity, surface area, and other factors [28,32]. The link between habitat complexity and fish assemblages has been well researched, with many studies showing positive relationships between complexity and fish abundance, biomass and diversity [27,33–38].

As mesophotic reefs often occur adjacent to inshore shallow reefs, some connectivity across the depth gradient might be expected. It was hypothesised in the late 1990s and early 2000s, for example, that mesophotic reefs provide refuge for some fish species [14,39,40]. This hypothesis assumed that mesophotic reefs were isolated from most of the stressors that impact inshore shallow reefs such as coral bleaching, pollution, habitat loss and some forms of fishing [40]. For temperate reef systems, there are insufficient data over sufficient temporal scales to make generalised conclusions about the extent or nature of any habitat connectivity between shallow and deep components. There is empirical evidence based on genetics and observations that connectivity between mesophotic and shallow reefs occurs, and this has been observed for over-exploited fishery target species [19,40]. On the other hand, there is also some evidence that mesophotic reefs are not merely extensions of shallow reefs, but host a unique species assemblage and would benefit from increased conservation management [3,4,41].

Surveys of mesophotic reefs have historically been logistically difficult and expensive due to the need for large offshore vessels and the lack of detailed information on their distribution and structure [29,42,43]. Earlier studies used coarse scale maps generated through the aggregation of information from commercial fishers, historical hydrographic data and targeted single beam acoustic surveys [44–46]. More recently, the expansion of swath acoustic surveys has resulted in high resolution maps of continental shelf rocky reefs based on the interpretation of bathymetry and backscatter [47,48]. The recent development of cost effective and easy to deploy underwater video equipment has also meant a move away from destructive survey methods such as gillnets, droplines and traps, which are also often not suitable for use in sensitive or protected areas. Baited remote underwater video (BRUV) is now commonly used to survey fish assemblages, and advances in camera housings, lights and study designs have enabled the deployment of cameras onto deeper habitats enabling non-destructive sampling of fishes across continental shelf waters [36,48–51]. Furthermore, there is evidence that BRUVs provide similar data when used at different depth and when compared to diver surveys [25,52].

While there has been some assessment of fish assemblages on shallow reefs in temperate eastern Australia [53–56], there has been no comparable assessment done on mesophotic reefs. The lack of knowledge on fish assemblages and habitat composition of mesophotic reefs in this region increases the uncertainties associated with marine spatial planning and evaluation of management effectiveness. This is particularly important in the case of marine parks that usually have specific objectives about conserving the biological diversity of a representative range of habitats and associated assemblages. Our study focussed on an area of the inner continental shelf within the Port Stephens-Great Lakes Marine Park (PSGLMP) and the adjacent Hunter Marine Park (HMP). The PSGLMP extends from the tidal limit to the 3 nm extent of State coastal waters, with the HMP extending from this boundary to 25 nm offshore. Specifically, the aim of our study was to quantify and compare the spatial distribution of fish assemblages on shallow (20–40 m) and lower-mesophotic (80–110 m) temperate rocky reefs, and relate these to habitat composition. This study will extend knowledge of temperate mesophotic reefs generally, but will also inform future decision-making in these two marine parks to improve effective management of these reefs and to design better targeted monitoring programs.

## Material and methods

### Study site

**Fig 1:**
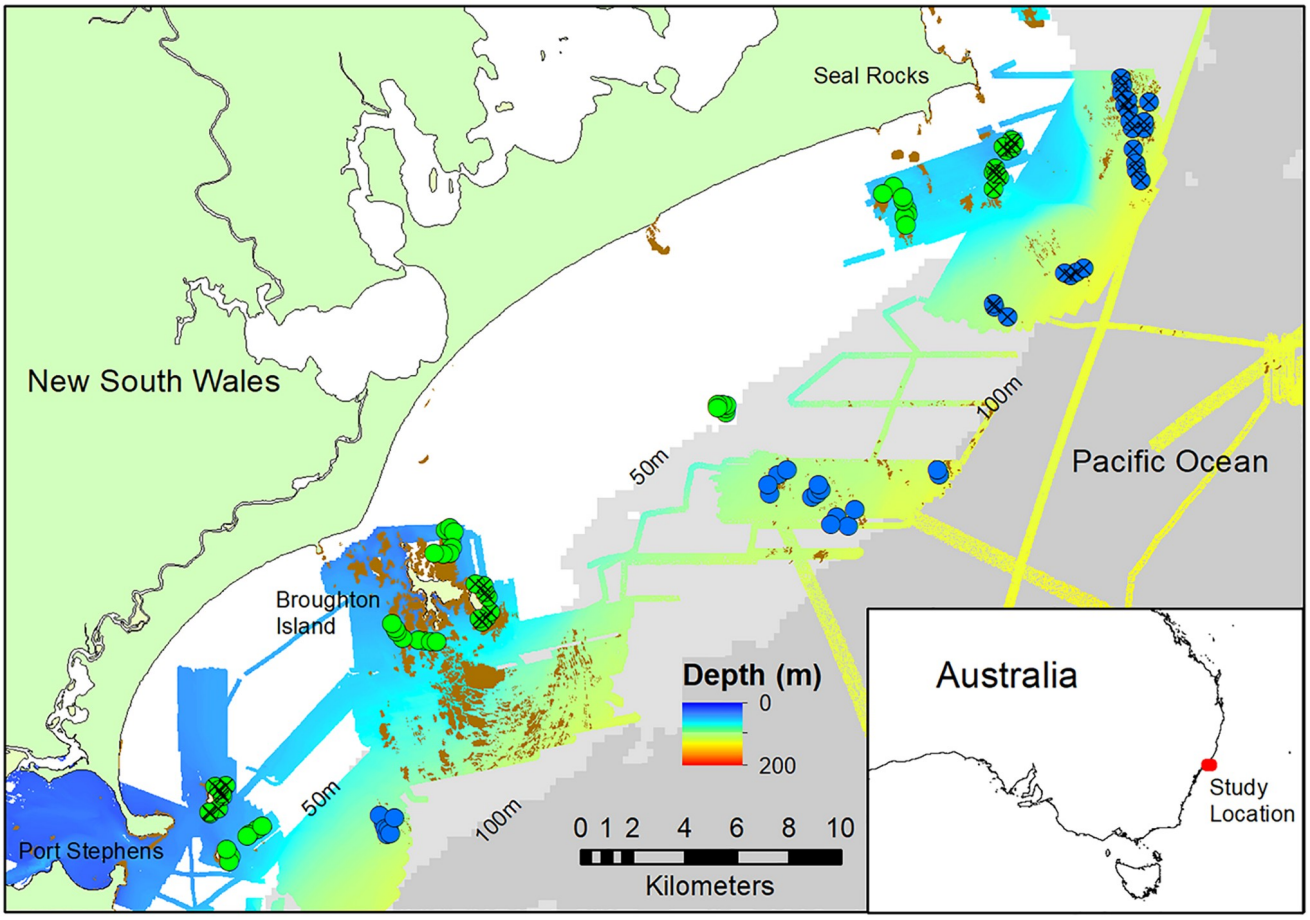
Map of the study area from Port Stephens to Seal Rocks on the east coast of Australia. The area that has been mapped using a swath acoustic sounder is indicated by the rainbow shaded area, while the 50m and 100m contours are represented by a differing shade of grey (source: GeoScience Australia). The brown shapes represent hand digitised reef with profile. Shallow stereo-BRUV deployments are delineated by the green circles and mesophotic stereo-BRUVs deployments are delineated by the blue circlers. Circles with a cross are stereo-BRUV deployments within a no-take area. Inset: Study location on the east coast of Australia.

This study took place along a ~40 km length of coastline between Port Stephens and Seal Rocks within the waters of the PSGLMP and HMP in New South Wales, Australia (-32°S; Fig 1). This region is dominated by temperate species, although some tropical vagrants often arrive in summer, reflecting the strong presence of the East Australian Current (EAC) that originates in the tropics [57–60]. This study was conducted during the austral spring (August to November) of 2016, a period of cool water influence in this region when surface temperatures are approximately 15–17°C.

Appropriate ethics (NSW Department of Primary Industries: ACEC REF 10_09) and fieldwork (NSW Department of Primary Industries research activities in NSW state waters: Permit No. P01/0059(A)-4.0 and research activities inside a marine park: Permit No. PSGLMP 2018/010) permits were obtained for this work.

### Reef mapping

In order to evaluate the extent, distribution and structure of rocky reefs in the study region, swath acoustic bathymetry and derived habitat data were collated from previous surveys [9]. In addition, targeted acoustic surveys were conducted in the region [61].

All acoustic surveys were conducted using a 125 kHz Geoswath interferometric swath system. Position and vessel motion for sonar acquisition was provided using a POS MV (Applanix, Canada) with Real-time Kinematic height and positional Virtual Reference Station corrections through SmartNet across the Telstra Mobile 3G network using Hypack (Hypack USA) acquisition software. Real-time ephemeris data were saved in POSPac log files for post-processing and calculating a 3 min forward-backward smooth for improved SBET in Single Base Station Mode. Mean SBET positional accuracies were improved to be better than 0.1 for X, Y and Z at nadir. Smoothed best estimates of trajectory were applied to Geoswath data before rough processing using amplitude, box, across-track and along-track filters in GS+. Data were exported as GSF for further data cleaning and cube modelling of soundings and production of a final digital elevation model. Backscatter data were output from GS+ in XTF data and then mosaiced using the Sidescan Solo module within Fledermaus FMGT. Reef extent was hand digitised from hillshaded bathymetry and derived slope layers and identified as ‘reef with profile’.

The Spatial Analyst tool and Benthic Terrain Modeller add-on in ArcGis v10.3.1 were used to analyse the cleaned bathymetric data. 50 m and 100 m radius buffers around individual stereo-BRUVS were used to calculate the mean, standard deviation and range for relief, rugosity, ruggedness, curvature and slope. Due to the 200 m separation between stereo-BRUVS, this ensured there were no overlaps. Pearson’s correlations were used to assess data obtained from the 50 m and 100 m radii for correlation.

### Sampling fish assemblage

Stereo baited remote underwater video (stereo-BRUV) was used to sample the fish assemblages at two depth strata, shallow reef (20-40m) and mesophotic reef (80-110m), as per the methodology set out in Langlois et al. [48]. Sampling sites were chosen using randomly selected grid references and a 1x1 km grid overlay on the plotted swath acoustic bathymetry maps. Each site consisted of four replicate stereo-BRUV deployments that were selected using 200x200 m grids to ensure each replicate was randomly selected yet spatially independent (a minimum of 200 m and maximum of 800 m between replicates). Sites were located within PSGLMP and the HMP, with 48 stereo-BRUV deployments located within no-take areas within the PSGLMP, and 59 stereo-BRUV deployments in areas that are fished. Hence, fishing status was included as a factor in the modelling (see Table 1).

**Table 1:** Description of factors used in both GAMM and RDA modelling.

Factor	Level / Range	Description
**Depth**	Shallow	Stereo-BRUV depth 20–40 m
	Mesophotic	Stereo-BRUV depth 80–110 m
**Fished**	Fished	Fishing allowed
	No-take	No fishing allowed
**Substrate**	Reef	100% reef in view
	Mixed	>50% reef, <50% sediment
	Sediment	>50% sediment, <50% reef
**Habitat**	Algae	Dominant habitat type is algae
	Algae sediment	Dominant habitat type is algae with sediment in view
	Invertebrates	Sessile invertebrate are dominant
	Invertebrates sediment	Sessile invertebrates are dominant with sediment in view
	Barrens	Urchin barrens, no algae, no sessile invertebrates
	Sediment	Field of view dominated by sediment
**Latitude**	-32.44–-32.71	The latitude of each stereo-BRUV deployment.
**Relief**	1.3–29.1	The range in bathymetry in the 50 m radius around each stereo-BRUV calculated in Spatial Analyst ArcGis
**Rugosity**	0.0–0.5	Arch-chord ratio rugosity index for the 50 m radius around each stereo-BRUV calculated in Spatial Analyst ArcGis
**Slope**	3.6–43.6	The rate of change in bathymetry for the 50 m radius around each stereo-BRUV calculated in Spatial Analyst ArcGis

Each deployment targeted rocky reef, and a deployment was considered successful if the stereo-BRUV landed on or immediately adjacent to rocky reef and when both the reef/benthos and water column could be viewed clearly. If a replicate was located over soft sediment it was moved to the nearest area of reef. Stereo-BRUVs were deployed for a period of 30 minutes which has been determined to be a sufficient time to obtain a representative sample of the fish community [62].

Each stereo-BRUV unit consisted of two Canon HG25 video cameras with a wide angle lens that were housed in two custom made SeaGIS Lty Ltd housings (http://www.seagis.com.au). Approximately one kilogram of pilchard (*Sardinops* sp.) was crushed in a plastic mesh bait bag and attached to the stereo-BRUV frame at the end of a 1.5 m long PVC pole. Due to the low light levels at depths >80m, we used Raytech subsea lights mounted to the centre of the stereo-BRUV frame at sites below that depth. A blue light was used as it is likely that the 450–465 nm wavelength is below the spectral sensitivity range of many fish species and therefore will have minimal effect on the fish behaviour [63]. On several occasions, white light was used to confirm identifications of fish species and to collect qualitative data on habitat type.

Video imagery collected by stereo-BRUVs was scored using standard metrics including scoring relative abundance (MaxN) as the maximum number of fish occurring in any one frame for each species. MaxN is widely accepted as the best method for estimating relative abundance from stationary video camera footage [49]. All fish were identified to the lowest taxonomic level possible, ideally to species level. For each stereo-BRUV deployment, the length of each *Chrysophrys auratus* (pink snapper) and *Nemadactylus douglasii* (blue morwong) observed at the time of MaxN was measured as total length (tip of fish nose to tip of the longest caudal lob). Total length was used as this is how the minimum legal length (MLL) is measured. The MLL for both of these species is 300 mm total length. These two species are considered fishery target species and are often used as indicator species in stereo-BRUV surveys [62]. All stereo-BRUV video analysis and scoring was done using the EventMeasure software (www.seagis.com). The video footage was also used to categorise substrate type and habitat type as factors in an attempt to relate species and species assemblage data to the environment and habitat (Table 1).

### Data analysis

To examine patterns in species assemblages, we used redundancy analysis (RDA). RDA is related to principal components analyses and is based on Euclidean distance, implying that each species is on an axis orthogonal to all other species, and sites are points in this multidimensional space [64]. Due to a number of schooling species occurring in high abundances, all species were Hellinger transformed before doing a forward stepwise model selection using a suite of explanatory factors (Table 1) to select the factors that best explained the dissimilarity in the species assemblage. The function “ordiR2step” from the “Vegan” package in R was used to select the most parsimonious model [65]. Permutation tests were used to test for the statistical significance of each marginal term. A triplot was used to visually determine and display the strength of the relationships between species assemblage and the explanatory factors that underpin the variation in species assemblage between stereo-BRUV deployments.

To investigate the spatial distribution of the fish assemblage across shallow and mesophotic reefs we used generalised additive mixed models (GAMMs). These can incorporate the non-linear patterns and overdispersion often encountered with spatially structured ecological studies. A suite of response variables was chosen *a priori* and these included species richness, total relative abundance, the most speciose families (Labridae, Monocanthidae and Carangidae) and species that are either abundant (*Pseudocaranx georgianus*, silver trevally and *Meuschenia scaber*, velvet leatherjacket) or of fishery interest (*C*. *auratus* and *N*. *douglasii*,). We also modelled the relative abundance of all recreationally and commercially targeted species pooled together. Recreationally targeted species were determined from West et al. [11], while commercially targeted species were selected from the assessment reports for the ocean trawl and trap & line fishery assessment reports [12]. Site, a cluster of four stereo-BRUV deployments, was used as the random factor.

Prior to any modelling, all data were explored using scatter and boxplots to assess for correlations between covariates and outliers in the response variables. To minimise the risk of overfitting any models, if two covariates had a Pearson’s correlation >0.7, then the variable that made the least ‘ecological sense’, according to the authors, in explaining the distribution of fish or was less replicable in future studies were removed.

A forward stepwise method was used to select the ‘best’ model based on Akaike information criterion (AIC). The first step ran models with individual predictor variables and the model with the lowest AIC was then selected. Step two ran models including the first predictor variable with all other variables and again selected the variable with the lowest AIC. This was repeated until the difference in the AIC was less than two. Models were limited to three predictor variables to minimise overfitting. Since GAMMs can account for data that are not normally distributed, models were fitted with untransformed data using a Poisson distribution. Once the final model had been decided, the model residuals were assessed for heterogeneity and overdispersion. If a model was considered overdispersed, the process was repeated but this time using the negative binomial distribution. Models with a negative binomial distribution were also assessed using a forward stepwise selection of the k value. All GAMM analyses were performed using the ‘GAMM4’ package in R [66].

The distribution of lengths for *C*. *auratus* and *N*. *douglassi* was investigated using boxplots and histograms. A Kolmogorov-Smirnoc two-sample test was used to compare the lengths distributions between shallow and mesophotic reef following the methods outlined in Langlois et al. [67]. This procedure was done use the ‘ks.boot’ function in the ‘Matching’ package in R [68]. We used 100,000 simulations to account for the small sample size.

## Results

### Summary of baited remote underwater video deployments

A total of 107 stereo-BRUVs were successfully completed, with 64 deployments on the shallow reef and 43 deployments on the mesophotic reef (Table 2). A total of 7368 individuals (sum of MaxN) from 96 species, representing 53 families were recorded (Table 2). A total of 79 species were recorded on shallow reef, of which 49 species were unique (Table 2). A total of 47 species were recorded on mesophotic reef, of which 17 species were unique (Table 2). Thirty species were found to occur on both shallow and mesophotic reef (Table 2).

**Table 2:** A summary of the number of stereo-BRUVs and species compositions recorded from stereo-BRUVs deployed on shallow and mesophotic reef.

	Shallow (20–40 m)	Mesophotic (80–110 m)
**No. of stereo-BRUV deployments**	64	43
**Species richness (SR)**	79	47
**Mean SR (± SE) per stereo-BRUV**	19(0.48)	9(0.47)
**Family richness**	42	35
**No. rare species**	26	17
**No. of species unique to reef depth**	49	17

Rare species were seen on fewer than three occasions.

Labridae and Monacanthidae were the most speciose families with nine species each, equating to 19% of the total species richness. On shallow reefs, *Ophthalmolepis lineolatus* (southern Maori wrasse) was the most ubiquitous species, being recorded on 100% of deployments, followed by *Notolabrus gymnogenis* (crimsonband wrasse, 94%) and *C*. *auratus* (92%). In comparison, on mesophotic reefs *Centroberyx affinis* (eastern nannygai) was the most ubiquitous species, being recorded on 74% of deployments, followed *by N*. *douglasii* at 72% and *Trachurus novaezelandiae* (yellowtail scad) at 60%. Two threatened species were also recorded, *Epinephelus daemelii* (black cod) on shallow reef and *Carcharias taurus* (grey nurse shark) on both shallow and mesophotic reef.

### Fish assemblage spatial distribution in relation to environment

**Fig 2:**
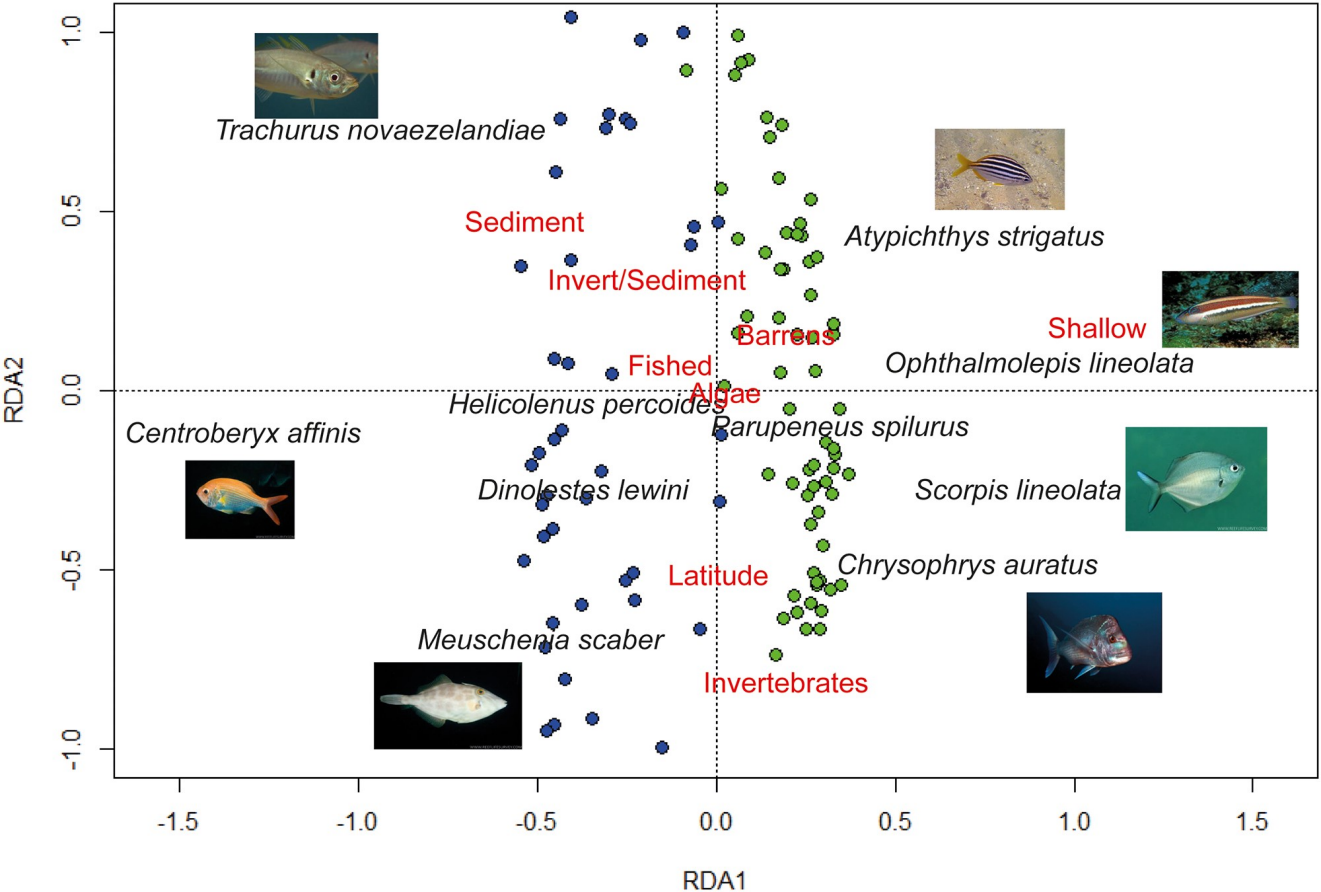
An RDA triplot ordination of transformed relative abundance data constrained by depth, latitude, fished/no-take. Filled circles represent mesophotic reef stereo-BRUV deployments and open circles represent shallow reef stereo-BRUV deployments.

The best fitting RDA model to describe the transformed species assemblage data included the factors depth, latitude, fished/no-take, habitat (Adj. R^2^ = 0.27, F = 5.82, P < 0.001). Permutation tests of each of these constraints gave significant marginal terms (depth: *F* = 30.35 *P* < 0.01, latitude: *F* = 4.01 *P* < 0.01, fished: *F* = 2.24 *P* = 0.02, habitat: *F* = 1.99 *P* < 0.01). The reef metrics relief, rugosity, ruggedness, curvature and slope were not significant in terms of explaining the transformed species assemblage data. The RDA ordination showed a clear division in stereo-BRUV deployments on shallow reefs and mesophotic reefs (Fig 2). The majority of shallow stereo-BRUV deployments had positive RDA1 values, while the majority of stereo-BRUV deployments on mesophotic reefs had negative RDA1 values (Fig 2). The RDA2 axis was mainly driven by habitat and latitude (Fig 2). Mesophotic reefs were characterised by the schooling species *T*. *novaezelandiae*, *C*. *affinis* and *M*. *scaber*, while shallow reefs were characterised by the schooling species *Atypichthys strigatus* (Australian mado) and *Scorpis lineolate* (silver sweep), as well the indicator species *C*. *auratus* and *O*. *lineolatus* (Fig 2).

### Species richness, relative abundance and family spatial distribution

**Fig 3:**
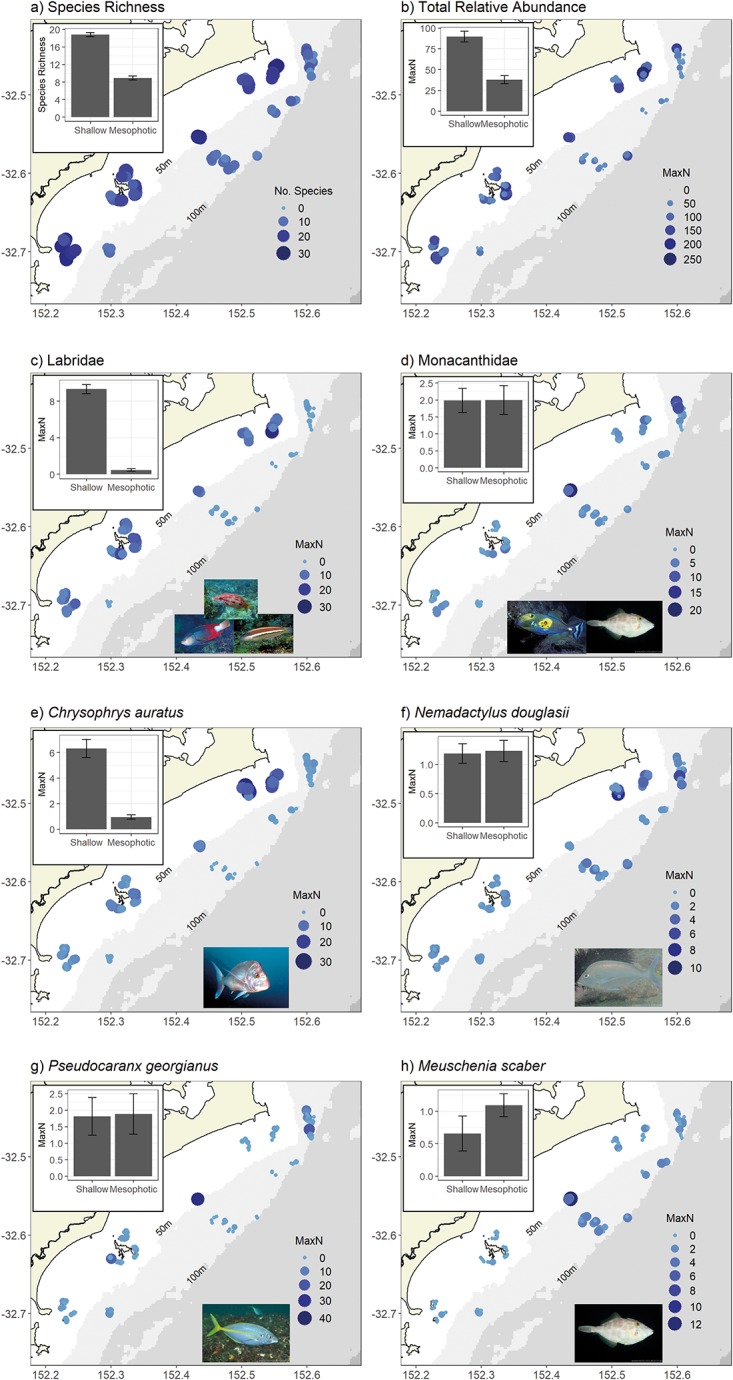
The spatial distribution of species richness, total MaxN, speciose families and species of interest. a) Distribution of species richness, b) total relative abundance, c) Labridae relative abundance, d) Monacanthidae relative abundance, e) *Chrysophrys auratus*, f) *Nemadactylus douglasii*, g) *Pseudocaranx georgianus* and h) *Meuschenia scaber* as observed by stereo-BRUVs across the study area. Bubble size and colour represents the species richness for each individual stereo-BRUV deployment. Inset plots: Mean (+/- SE) of species richness and MaxN for shallow and mesophotic stereo-BRUV deployments.

The species richness recorded on shallow reef stereo-BRUVs was nearly double that recorded on mesophotic reef stereo-BRUVs, with little variation between deployments (Fig 3). The most parsimonious GAMM for species richness included the factors depth, substrate and rugosity (Table 3). Stereo-BRUVs that landed on top of rocky reef had the highest species richness, with a significant positive relationship with rugosity. The total relative abundance (total MaxN) of all fishes followed a similar pattern, with more than double the number of fishes recorded on shallow reefs compared with mesophotic reefs (Fig 3). Depth and habitat provided the most parsimonious GAMM model (Table 3). Sites that were dominated by urchin barrens and sediment habitats had the highest total MaxN. This pattern was driven by the high numbers of schooling species of fish such as *A*. *strigatus* and *T*. *novaezelandiae* that commonly occurred across the shallow reefs.

**Table 3:** The best GAMMs for predicting, species richness, relative abundance of key families, relative abundance of most targeted species and all targeted species pooled.

Dependent variable		d.f.	Adj. R^2^	AIC	BIC	Best model
**Species richness**		5.95	0.736	485.5	503.1	Depth, Rugosity, Substrate
Total relative abundance*		7	0.305	904.7	927.3	Depth, Habitat
**Family**	Labridae	5.14	0.715	371.5	384.1	Depth, Rugosity
	Monacanthidae	6	0.042	326.4	344	Habitat
	Carangidae*	13.41	0.224	552.4	580	Habitat, Relief, Slope
**Species**	*Chrysophrys auratus*	7.57	0.547	396.7	419.3	Depth, Latitude, Habitat
	*Nemadactylus douglasii*	7	0.118	263.57	283.66	Latitude, Habitat
	*Psuedocaranx georgianus*	10.57	0.324	293.1	318.2	Slope, Rugosity, Habitat
	*Meuschenia scaber*	4.9	0.531	228.5	252.4	Depth, Habitat, Latitude
**All fishery targeted species**		16.7	0.210	817.9	845.4	Habitat, Rugosity, Slope

* negative binomial model. BIC = Bayesian information criterion.

The family Labridae was much more abundant on shallow reefs compared to mesophotic reefs (Fig 3). All nine species of Labridae were recorded on shallow reefs, with *O*. *lineolatus* and *Coris Picta* (comb wrasse) being the most relatively abundant. Only one species of Labridae, *Bodianus unimaculatus* (eastern pigfish), was recorded on mesophotic reefs. The family Monacanthidae were more equally distributed across reef type (Fig 3), with all nine species occurring on the shallow reefs, while three species were recorded on both reef types. *M*. *scaber* was the most abundant species on both reef types. The best model for Monacanthidae included the single factor habitat (Fig 3, Table 3). Habitats dominated by a high abundance of sessile invertebrates had the highest relative abundance of monacanthids.

The distribution of fishery targeted species across the two depth categories was highly variable and species dependent. The spatial distribution of the most targeted species (*C*. *auratus*) was related to depth, latitude and habitat type (Table 3). On average, the relative abundance of *C*. *auratus* on shallow reefs was six times greater than on mesophotic reefs (Fig 3), although, there was greater variability between stereo-BRUV deployments on shallow reefs (Fig 3). The positive effect of latitude showed that abundances of *C*. *auratus* were highest at the Seal Rocks sites, the most northern survey site (Fig 3). Also, *C*. *auratus* occurred in greater abundance on stereo-BRUV deployments that were located on the edge of reefs that were dominated by invertebrate/sediment or sediment habitats. *N*. *douglasii*, also a highly targeted species, was more evenly distributed across reef type, with latitude and habitat type providing the best model to describe the spatial distribution of this species (Table 3). The positive relationship between latitude and relative abundance of *N*. *douglasii* showed greater abundance at Seal Rocks sites (Fig 3). The spatial distribution of the carangid *P*. *georgianus* was far more variable than *C*. *auratus* and *N*. *douglasii*. However, on average the relative distribution was similar across the two depth categories (Fig 3). *P*. *georgianus* tend to be observed in small schools on low rugosity reef edges. The most parsimonious model that best described the spatial distribution of *P*. *georgianus* included the factors slope, rugosity and habitat (Table 3). The spatial distribution of the monacanthid *M*. *scaber* was highly variable, but on average higher relative abundances were observed on mesophotic reefs (Fig 3). The most parsimonious model best described the spatial distribution of *M*. *scaber* included the factors depth, habitat and latitude (Table 3). It was sites within the mid latitudes of this study that had the highest relative abundances and the higher relative abundances tended to be on low relief reef (Fig 3).

**Fig 4:**
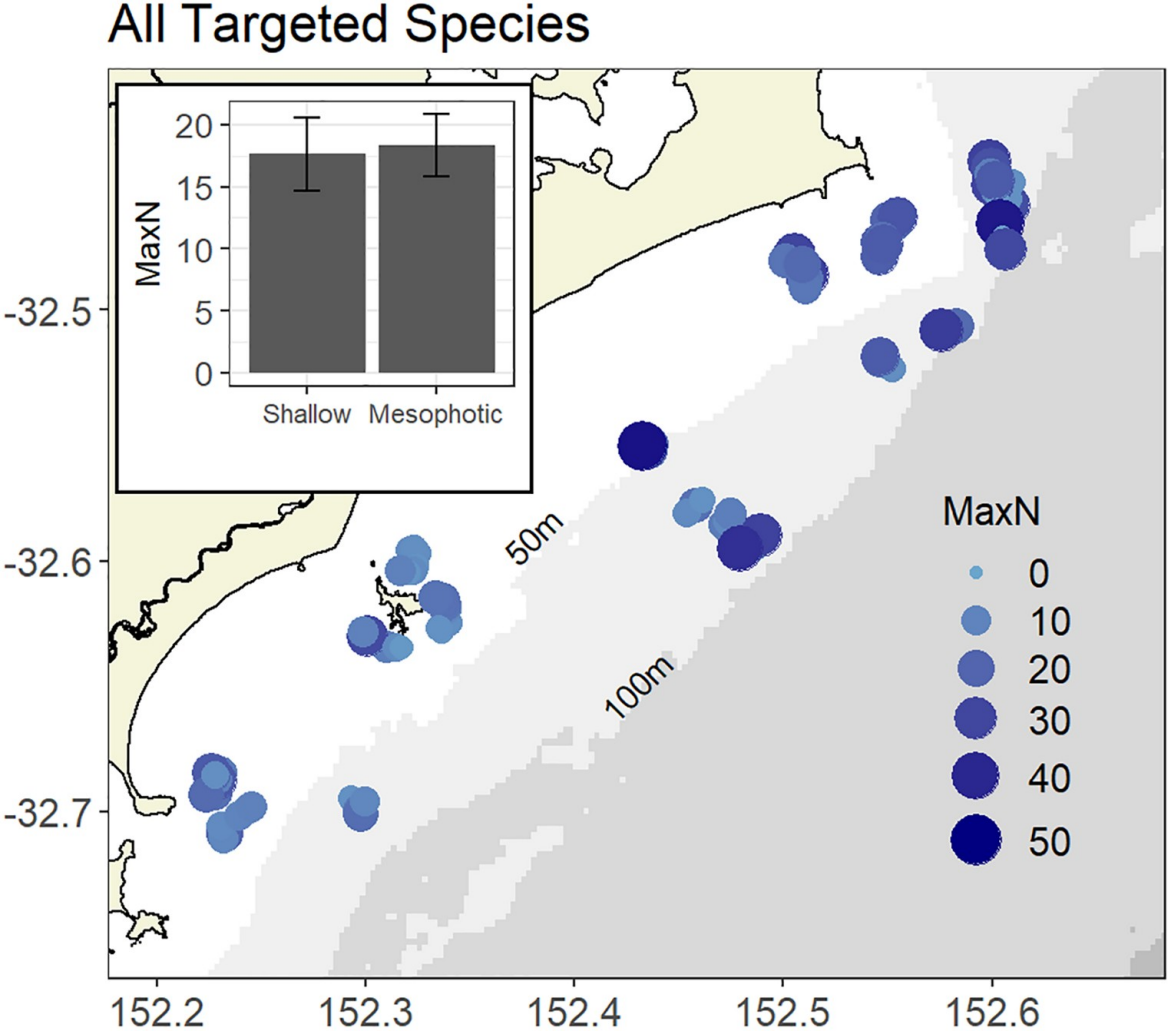
The spatial distribution of fishery targeted species. Distribution of all commercial and recreationally targeted species as observed by stereo-BRUVs across the study area. Bubble size and colour represents the MaxN of all commercial and recreationally targeted species for each individual stereo-BRUV deployment. Inset plot: Mean (+/- SE) MaxN of all commercial and recreationally targeted species across shallow and mesophotic reef.

Species that are actively targeted and highly retained by both recreational and commercial fishers showed a relatively equal distribution across both shallow and mesophotic reefs (Fig 4). Habitat, rugosity and slope best described the variability between sites (Table 3). Reef dominated by algae and reef edge habitats had the highest abundance of fishery-targeted species. While there was a strong positive relationship between fishery-targeted species and reef rugosity, there was a weak negative relationship with slope.

### Length distribution of *C*. *auratus* and *N*. *doulgassi*

**Fig 5:**
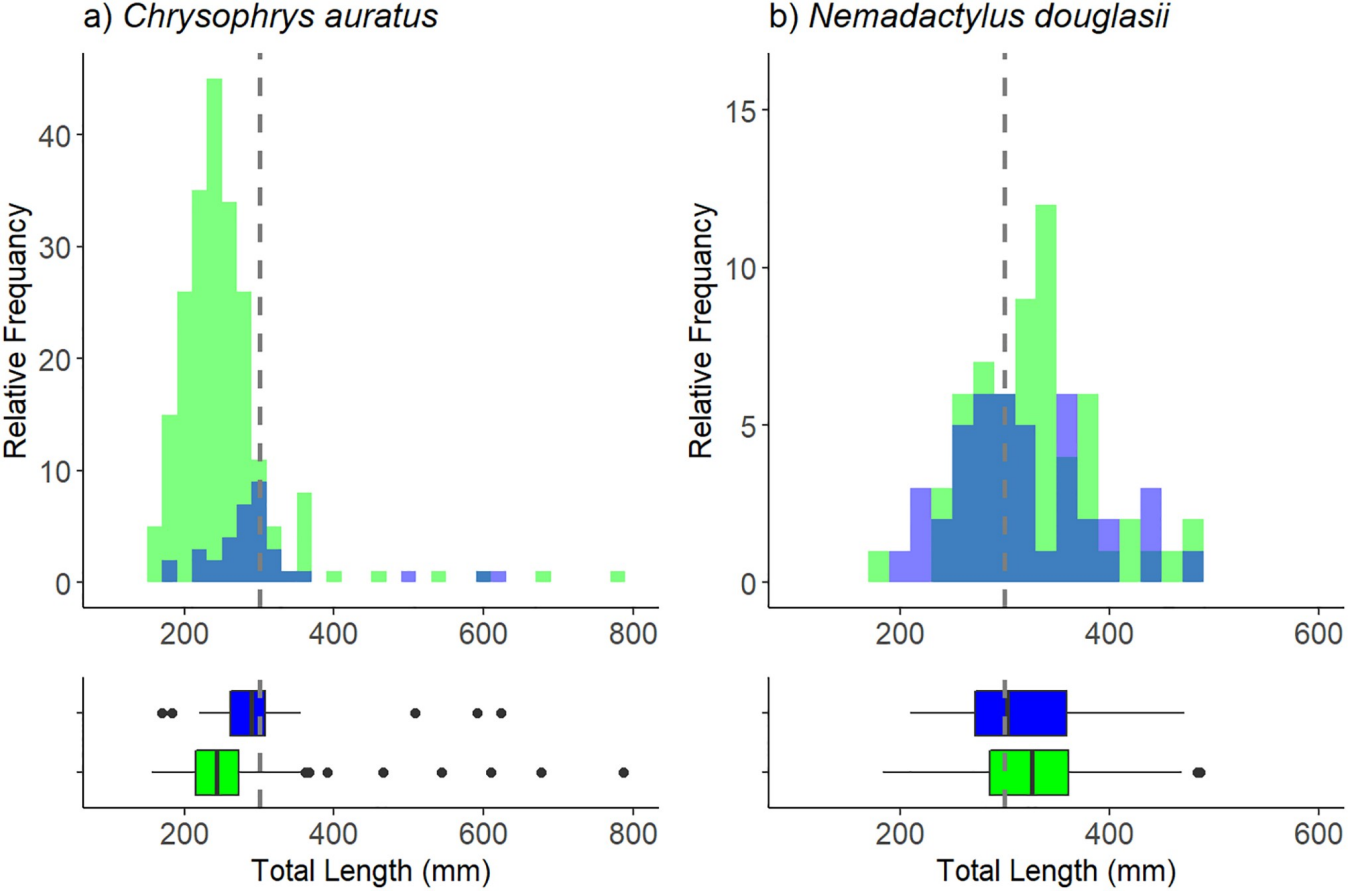
Lengths of two of the prominent fishery targeted species. a) the length distribution histogram for the total lengths of *C*. *auratus* at shallow (green) and mesophotic (blue) depths. A boxplot below summarises the distribution of total lengths for *C*. *auratus* at shallow (green) and mesophotic (blue) depths. b) The length distribution histogram for the total lengths of *N*. *douglassi* at shallow (green) and mesophotic (blue) depths. Boxplots below summarise the distribution of total lengths for *N*. *douglassi* at shallow (green) and mesophotic (blue) depths. The MLL for each species has been indicated by a dashed line on each plot.

The mean length of *C*. *auratus* recorded on mesophotic reefs was larger than those recorded on shallow reef, with the mean total length of fish on mesophotic reef slightly below the MLL for retention of this species (Fig 5a). Mesophotic reefs also had a greater proportion of legally sized fish at 48% compared to shallow reefs 13%. The distribution of lengths also varied between shallow and mesophotic reef with *C*. *auratus* at both depths having a unimodal distribution. However, the mode length on mesophotic reef was greater than that recorded on shallow reef (Fig 5a). A Kolmogorov–Smirnov test comparing the length distributions between depths rejected the null hypothesis, suggesting there was a significant difference in the distribution of *C*. *auratus lengths* (D = 0.46, *p* = <0.001; Fig 5a).

The mean lengths of *N*. *douglassi* were very similar between mesophotic and shallow reef (Fig 5b). The proportion of fish above the MLL for *N*. *douglassi* was similar for at both depths. The length distributions appeared to be bi-modal and at both shallow and mesophotic reef with the modes not overlapping (Fig 5b). However, Kolmogorov–Smirnov tests comparing lengths at the two depth categories for *N*. *douglassi* showed no significant difference between depth categories (D = 0.20, *p* = 0.19; Fig 5b).

## Discussion

The fish assemblages on rocky reefs at lower-mesophotic depths (80–110 m) were found to be distinct from those associated with adjacent shallow rocky reefs (20–40 m). Despite the large differences in species richness and relative abundance of all fishes, 30 species (i.e. 31% all species recorded during this study) occurred across the depth categories that were sampled. This is one of the first studies to compare rocky reef fish assemblages across the 80–90 m range in depths at these latitudes, and there are few comparable temperate studies. The vast majority of mesophotic reef research has occurred in tropical systems in Australia, the USA and Caribbean [18]. There are even fewer studies (~1%) using BRUVs, particularly stereo-BRUVs, to sample fish and habitats at mesophotic depths [18]. The species richness that we recorded from stereo-BRUVs on these temperate mesophotic reefs is approximately half of what has been recorded using stereo-BRUVs in tropical systems [4,14,51]. However, the species richness to depth gradient relationship is consistent across both tropical and temperate systems as noted in previous studies [4,69], as well as the current study.

The transition zone between the shallow and mesophotic species assemblages is unclear in this study as we did not sample between depths of 40–80 m. The mesophotic reef that was sampled during this study is disconnected from rocky reefs found in shallow waters as it is separated by large expanses of soft sediment habitats [61]. The current referenced global definition for the transition to mesophotic ecosystems is 40 m [16,70], but this will be regionally specific and dependent on factors such as light, temperature and habitat [18]. The connectivity across the depth gradient is also a significant unknown. An understanding of depth connectivity is needed to determine if mesophotic reefs can provide refuge from short-term pressures such as storms or heatwaves or long-term pressures such as fishing. There is evidence that mesophotic reefs can provide ‘refuge’ habitat for fishery-targeted species [14,39,40,71]. Some caution is needed when making generalisations of connectivity across depths ranges, however, as there is evidence of differing life histories and intraspecific variability in demographic traits of fishes using reefs at mesophotic depths [72].

This study found strong depth related patterns that could be coupled with depth related reef and habitat complexity [4,6,26]. In all but two models (Species richness and Labridae), the factor habitat was selected, highlighting the importance of habitat in describing the distribution of fishes. The correlation between increasing depth and decreasing light equates to a change in habitat structure, with the shallower reefs dominated by macroalgae [73,74], and at depths >30m sessile invertebrates, such as sponges and octocorals. As the shallow reefs surveyed in this study were in 20–40 m depth range, a transition between algae and sessile invertebrate dominated habitats was observed. Therefore, herbivorous and omnivorous fishes are more likely to inhabit shallow reefs where algae is present, thus increasing species richness and abundance. In comparison, at mesophotic depths it is expected that carnivorous, planktivorous, and scavenger fish and species with greater tolerances to ocean currents and thermoclines would occur. These finding are consistent with many tropical mesophotic coral ecosystem studies particularly in the Caribbean and the Indo-Pacific region [3,41,52].

Apart from light availability and habitat, ocean currents and temperature gradients or thermoclines further separate mesophotic reefs from adjacent shallow reefs [4]. In temperate Eastern Australia, the EAC has the greatest influence on the oceanography and connectivity of deeper reefs [75,76]. This is particularly relevant as the strength and the seasonality of the EAC has recently changed with warmer water pushing further south and for longer periods of time [77]. As the EAC is at its strongest (fastest flowing and warmer temperatures) across these mesophotic reefs, the EAC has the potential to influence range extension or a change in the distribution of fishes. However, the majority of knowledge on the changing EAC is based on surface waters and there is a knowledge gap of how currents and water temperatures are changing at depth in the EAC off the NSW coastline. Therefore, the EAC has the potential to influence biodiversity, abundance of fishery targeted species and species of conservation significance [69]. Further seasonal sampling is required to test hypotheses about the effects of seasonality and the EAC, i.e. differences between warm and cold water periods.

The physical structure of the reefs (rugosity, slope and relief) is likely to have the greatest influence on the spatial distribution of fishes on rocky reefs. The swath acoustic data were beneficial in selecting reefs to sample, but they can also be used to derive metrics that can possibly predict the spatial distribution of species richness, species of interest and all fishery target species pooled together. The derivation of habitat metrics from swath acoustic data can provide various levels of explanatory or predictive ability relating to fish assemblage composition and distribution [27,31,34,78,79]. In this study, rugosity, slope or relief were selected in the ‘best’ models explaining the variability in many aspects of the fish assemblages that were sampled. While many studies focus on explaining or predicting the spatial distribution of individual species, a more relevant application for spatial planning would be to use reef metrics to explain or predict at higher levels such as species richness or pooled fishery targeted species. For this study, a combination of depth, substrate type, habitat type and rugosity best described species richness and fishery targeted species. These two variables could provide managers with information on areas of high fish diversity or fishery significance.

At a species level, the explanatory factors varied among the four species that were selected a priori for analysis. *C*. auratus is arguably the most important recreational and commercial fishery in this region [55]. While *C*. *auratus* were twice as abundant on shallow reef, on average *C*. *auratus* were larger around mesophotic reefs. In proportion, there were more *C*. *auratus* above the MLL for retention and thus considered sexually mature [80] on mesophotic reefs. This supports the hypothesis that shallow inshore reefs provide important habitat for juvenile *C*. *auratus*, while deeper mesophotic reef provide additional habitat for larger mature *C*. *auratus*. This is possibly due to either or both ontogenetic movement of larger fish to deeper waters or localised fishing pressure removing larger fish from shallow waters [15,25,81,82]. An ontogenetic change in habitat has been associate with habitat, prey and temperature [82,83]. Larger fish are often more tolerant to colder water and change their diets to organisms that are more abundant at deeper depths [82,84]. Unfortunately, data on fishing pressure in this area are limited and it is difficult to make inferences about the effects of fishing pressure on the size structure. A recent study did find that *C*. *auratus* are larger in no-take zones indicating fishing pressure does have some impact on the size structure of a localised population [55,56]. In general, *C*. *auratus* are known to have relatively small home ranges, but some individuals move hundreds of kilometres [85–87]. This has been demonstrated in the PSGLMP where tagged *C*. *auratus* demonstrated small home ranges with strong site fidelity on shallow reefs [85], but their movements and use of deeper reef habitats is unknown and warrants further investigation.

In contrast, the distribution of *N*. *douglassi* was more influenced by latitude than by depth. They do occur across a range of habitats including soft sediment and rocky reef and mainly feed on soft sediment associated molluscs and crustaceans [88,89]. *N*. *douglassi* are medium to large bodied fish that are targeted by recreational and commercial fishers. On average, the *N*. *douglassi* recorded during this study were above the MLL for retention and could be considered sexually mature [89]. On this section of coastline the relative abundance and lengths of *N*. *douglassi* are fairly constant across the depth gradient. Similarly, *P*. *dentex* was commonly observed on both types of reef, but they were patchier in their distribution. They were often observed in large schools on stereo-BRUV deployments positioned on the edge of reef or over adjacent soft-sediments. They too are targeted and retained by recreational and commercial fishers [89]. *M*. *scaber* is the most numerically abundant monacanthid species in eastern Australia and New Zealand, but very little is known of its ecology and biology [90]. They are known to inhabit a wide range of depths, but in this study there appears to be a preference for deeper mesophotic reefs. Feeding exclusively on sessile invertebrates such as sponges, ascidians, polyzoans, hydroids and barnacles, *M*. *scaber* is well suited to these mesophotic reefs [90]. For *N*. *douglassi* and *P*. *dentex* there doesn’t appear to be any preference for a particular depth of reef and nor is there any clear ecological rationale for one.

This study demonstrated that the fish assemblages of rocky reef at mesophotic depths are statistically different to the adjacent shallow reef systems. Despite more than double the total abundances, there were similar relative abundances of fishery target species across both shallow and mesophotic reefs suggesting that, from a fisheries management perspective, these reef systems have the potential for similar social and economic values. Increased knowledge and access to improved technology is now allowing boat-based recreational fishers to target deeper reefs. Swath acoustic data contributed to explaining the spatial distribution of each aspect of the fish assemblage. There is still a research need to investigate seasonal patterns and fine-scale intra-reef variability in fish assemblages on these temperate mesophotic reefs. The use of a complementary method, such as remotely operate vehicle or towed video that passively samples the fish assemblages would provide valuable information on the species not captured through the use of stereo-BRUV sampling [91]. Notwithstanding this limitation, this study has clearly demonstrated that reefs at mesophotic depths are important and should be taken more into consideration by both fishery managers and when zoning marine parks.

## References

[1] Morueta-Holme N, Enquist BJ, McGill BJ, Boyle B, Jørgensen PM, Ott JE, et al Habitat area and climate stability determine geographical variation in plant species range sizes. Ecol Lett. 2013;16: 1446–1454. 10.1111/ele.12184 24119177 PMC4068282

[2] Brown JH, Stevens GC, Kaufman DM. The geographic range: size, shape, boundaries, and internal structure. Annu Rev Ecol Syst. Annual Reviews. 1996;27: 597–623.

[3] Brokovich E, Einbinder S, Shashar N, Kiflawi M, Kark S. Descending to the twilight-zone: changes in coral reef fish assemblages along a depth gradient down to 65 m. Mar Ecol Prog Ser. 2008;371: 253–262.

[4] Sih TL, Cappo M, Kingsford M. Deep-reef fish assemblages of the Great Barrier Reef shelf-break (Australia). Sci Rep. 2017;7: 10886 10.1038/s41598-017-11452-1 28883506 PMC5589835

[5] Malcolm HA, Jordan A, Smith SDA. Biogeographical and cross-shelf patterns of reef fish assemblages in a transition zone. Mar Biodivers. 2010;40: 181–193.

[6] Bridge T, Done TJ, Friedman A, Beaman RJ. Variability in mesophotic coral reef communities along the Great Barrier Reef, Australia. Mar Ecol Prog Ser. 2011;428: 63–75.

[7] Kahng SE, Garcia-Sais JR, Spalding HL, Brokovich E, Wagner D, Weil E, et al Community ecology of mesophotic coral reef ecosystems. Coral Reefs. 2010;29: 255–275.

[8] Choat JH, Schiel DR. Patterns of distribution and abundance of large brown algae and invertebrate herbivores in subtidal regions of northern New Zealand. J Exp Mar Bio Ecol. 1982;60: 129–162.

[9] Jordan A, Davies P, Ingleton T, Foulsham E, Neilson J, Pritchard T. Seabed habitat mapping of the continental shelf of NSW. NSW Office of Environment and Heritage; 2010. https://www.environment.nsw.gov.au/-/media/OEH/Corporate-Site/Documents/Research/Our-science-and-research/seabed-habitat-mapping-continental-shelf-nsw-101057.pdf

[10] Terlizzi A, Anderson MJ, Fraschetti S, Benedetti-Cecchi L. Scales of spatial variation in Mediterranean subtidal sessile assemblages at different depths. Mar Ecol Prog Ser. 2007;332: 25–39.

[11] West LD, Stark KE, Murphy JJ, Lyle JM, Ochwada-Doyle FA. Survey of recreational fishing 2013/14. NSW Department of Primary Industries; 2015 Dec. Report No. 149. https://www.dpi.nsw.gov.au/__data/assets/pdf_file/0011/598628/West-et-al-Survey-of-rec-fishing-in-NSW-ACT-2013-14-2016_03_02.pdf

[12] New South Wales Department of Primary Industries. Assessment of the NSW ocean trawl fishery; prepared for the Department of the Environment and Energy for the purpose of assessment under Part 13 and 13(A) of the Environment Protection and Biodiversity Act 1999 [Internet]. NSW Department of Primary Industries; 2017 Sep. https://www.environment.gov.au/system/files/consultations/7adc92d2-f110-4f8f-8679-d4f6a5d39b8c/files/nsw-ocean-trawl-fishery-submission-2017.pdf

[13] Bo M, Bava S, Canese S, Angiolillo M, Cattaneo-Vietti R, Bavestrello G. Fishing impact on deep Mediterranean rocky habitats as revealed by ROV investigation. Biol Conserv. 2014;171: 167–176.

[14] Lindfield SJ, Harvey ES, Halford AR, McIlwain JL. Mesophotic depths as refuge areas for fishery-targeted species on coral reefs. Coral Reefs. 2016;35: 125–137.

[15] Pinheiro HT, Goodbody-Gringley G, Jessup ME, Shepherd B, Chequer AD, Rocha LA. Upper and lower mesophotic coral reef fish communities evaluated by underwater visual censuses in two Caribbean locations. Coral Reefs. 2016;35: 139–151.

[16] Hinderstein LM, Marr JCA, Martinez FA, Dowgiallo MJ, Puglise KA, Pyle RL, et al Theme section on mesophotic coral ecosystems: characterization, ecology, and management. Coral Reefs. 2010;29: 247–251.

[17] Baker EK, Puglise KA, Harris PT. Mesophotic coral ecosystems—A lifeboat for coral reefs? The United Nations Environment Programme and GRID-Arendal, Nairobi and Arendal; 2016.

[18] Turner JA, Babcock RC, Hovey R, Kendrick GA, Degraer S. Deep thinking: a systematic review of mesophotic coral ecosystems. ICES J Mar Sci. 2017;74: 2309–2320.

[19] Loya Y, Eyal G, Treibitz T, Lesser MP, Appeldoorn R. Theme section on mesophotic coral ecosystems: Advances in knowledge and future perspectives. Coral Reefs. 2016;35: 1–9.

[20] Lucieer V, Porter-Smith R, Nichol S, Monk J, Barrett N. Collation of existing shelf reef mapping data and gap identification: Phase 1 final report—Shelf reef key ecological features. Marine Biodiversity Hub, University of Tasmania 2016.

[21] Nichol S, Huang Z, Howard F, Porter-Smith R, Lucieer V, Barrett N. Geomorphological classification of reefs—Draft framework for an Australian standard. Marine Biodiversity Hub, GeoScience Australia 2016.

[22] Rooney J, Donham E, Montgomery A, Spalding H, Parrish F, Boland R, et al Mesophotic coral ecosystems in the Hawaiian Archipelago. Coral Reefs. 2010;29: 361–367.

[23] de Oliveira Soares M, Davis M, de Paiva CC, de Macêdo Carneiro PB. Mesophotic ecosystems: coral and fish assemblages in a tropical marginal reef (northeastern Brazil). Mar Biodivers. 2016; 1–6.

[24] Harris PT, Heap AD, Marshall JF, McCulloch M. A new coral reef province in the Gulf of Carpentaria, Australia: Colonisation, growth and submergence during the early Holocene. Mar Geol. 2008;251: 85–97.

[25] Heyns-Veale ER, Bernard ATF, Richoux NB, Parker D, Langlois TJ, Harvey ES, et al Depth and habitat determine assemblage structure of South Africa’s warm-temperate reef fish. Mar Biol. 2016;163: 158.

[26] Englebert N, Bongaerts P, Muir PR, Hay KB, Pichon M, Hoegh-Guldberg O. Lower mesophotic coral communities (60–125 m depth) of the northern Great Barrier Reef and Coral Sea. PLoS One. 2017;12: e0170336 10.1371/journal.pone.0170336 28146574 PMC5287465

[27] Rees MJ, Jordan A, Price OF, Coleman MA, Davis AR. Abiotic surrogates for temperate rocky reef biodiversity: implications for marine protected areas. Divers Distrib. 2014;20: 284–296.

[28] Collins DL, Langlois TJ, Bond T, Holmes TH, Harvey ES, Fisher R, et al A novel stereo-video method to investigate fish–habitat relationships. Methods Ecol Evol. 2017;8: 116–125.

[29] Cameron MJ, Lucieer V, Barrett NS, Johnson CR, Edgar GJ. Understanding community-habitat associations of temperate reef fishes using fine-resolution bathymetric measures of physical structure. Mar Ecol Prog Ser. 2014;506: 213–229.

[30] Connell SD, Kingsford MJ. Spatial, temporal and habitat-related variation in the abundance of large predatory fish at One Tree Reef, Australia. Coral Reefs. 1998;17: 49–57.

[31] Rees MJ, Knott NA, Neilson J, Linklater M, Osterloh I, Jordan A, et al Accounting for habitat structural complexity improves the assessment of performance in no-take marine reserves. Biol Conserv. 2018;224: 100–110.

[32] Beck MW. Separating the elements of habitat structure: independent effects of habitat complexity and structural components on rocky intertidal gastropods. J Exp Mar Bio Ecol. 2000;249: 29–49. 10817826 10.1016/s0022-0981(00)00171-4

[33] Cappo M, Speare P, De’ath G. Comparison of baited remote underwater video stations (BRUVS) and prawn (shrimp) trawls for assessments of fish biodiversity in inter-reefal areas of the Great Barrier Reef Marine Park. J Exp Mar Bio Ecol. 2004;302: 123–152.

[34] Pittman SJ, Brown KA. Multi-scale approach for predicting fish species distributions across coral reef seascapes. PLoS One. 2011;6: e20583 10.1371/journal.pone.0020583 21637787 PMC3102744

[35] Harman N, Harvey ES, Kendrick GA. Differences in fish assemblages from different reef habitats at Hamelin Bay, south-western Australia. Mar Freshwater Res. 2003;54: 177–184.

[36] McLean DL, Langlois TJ, Newman SJ, Holmes TH, Birt MJ, Bornt KR, et al Distribution, abundance, diversity and habitat associations of fishes across a bioregion experiencing rapid coastal development. Estuar Coast Shelf Sci. 2016;178: 36–47.

[37] Harvey ES, Cappo M, Kendrick GA, McLean DL. Coastal fish assemblages reflect geological and oceanographic gradients within an Australian zootone. PLoS One. 2013;8: e80955 10.1371/journal.pone.0080955 24278353 PMC3838414

[38] Ferreira CEL, Goncçalves JEA, Coutinho R. Community structure of fishes and habitat complexity on a tropical rocky shore. Environ Biol Fishes. 2001;61: 353–369.

[39] MacDonald C, Bridge T, Jones GP. Depth, bay position and habitat structure as determinants of coral reef fish distributions: Are deep reefs a potential refuge? Mar Ecol Prog Ser. 2016;561: 217–231.

[40] Thomas CJ, Bridge TCL, Figueiredo J, Deleersnijder E, Hanert E. Connectivity between submerged and near-sea-surface coral reefs: can submerged reef populations act as refuges? Diversity and Distributions. 2015;21: 1254–1266.

[41] Bejarano I, Appeldoorn RS, Nemeth M. Fishes associated with mesophotic coral ecosystems in La Parguera, Puerto Rico. Coral Reefs. 2014;33: 313–328.

[42] Armstrong RA, Singh H. Mesophotic coral reefs of the Puerto Rico Shelf In: Harris PT, Baker EK, editors. Seafloor geomorphology as benthic habitat. London: Elsevier; 2012 pp. 365–374.

[43] Trembanis AC, Forrest AL, Keller BM, Patterson MR. Mesophotic coral ecosystems: a geoacoustically derived proxy for habitat and relative diversity for the leeward shelf of Bonaire, Dutch Caribbean. Frontiers in Marine Science. 2017;4: 51.

[44] Bax NJ, Williams A. Seabed habitat on the south-eastern Australian continental shelf: context, vulnerability and monitoring. Mar Freshwater Res. 2001;52: 491–512.

[45] Bax N, Kloser R, Williams A, Gowlett-Holmes K, Ryan T. Seafloor habitat definition for spatial management in fisheries: A case study on the continental shelf of southeast Australia. Oceanol Acta. 1999;22: 705–720.

[46] Williams A, Bax NJ. Delineating fish-habitat associations for spatially based management: an example from the south-eastern Australian continental shelf. Mar Freshwater Res. 2001;52: 513–536.

[47] Lecours V, Devillers R, Schneider DC, Lucieer VL, Brown CJ, Edinger EN. Spatial scale and geographic context in benthic habitat mapping: review and future directions. Mar Ecol Prog Ser. 2015;535: 259–284.

[48] Langlois T, Williams J, Monk J, Bouchet P, Currey L, Goetze J, et al. Marine sampling field manual for benthic stereo BRUVs (baited Remote underwater videos) [Version 1]. NESP Marine Biodiversity Hub; 2018;

[49] Cappo M, Harvey E, Shortis M. Counting and measuring fish with baited video techniques—an overview. In: Lyle J, Furlani DM, Buxton CD, editors. Proceedings of the 2006 Australian Society of Fish Biology conference and workshop cutting edge technologies in fish and fisheries science. ASFB; 2007. pp. 101–114.

[50] Whitmarsh SK, Fairweather PG, Huveneers C. What is big BRUVver up to? Methods and uses of baited underwater video. Rev Fish Biol Fish. 2017;27: 53–73.

[51] Hill NA, Barrett N, Lawrence E, Hulls J, Dambacher JM, Nichol S, et al Quantifying fish assemblages in large, offshore marine protected areas: an Australian case study. PLoS One. 2014;9: e110831 10.1371/journal.pone.0110831 25360763 PMC4215995

[52] Andradi-Brown DA, Gress E, Wright G, Exton DA, Rogers AD. Reef fish community biomass and trophic structure changes across shallow to upper-mesophotic reefs in the Mesoamerican Barrier Reef, Caribbean. PLoS One. 2016;11: e0156641 10.1371/journal.pone.0156641 27332811 PMC4917088

[53] Malcolm HA, Schultz AL, Sachs P, Johnstone N, Jordan A. Decadal changes in the abundance and length of snapper (Chrysophrys auratus) in subtropical marine sanctuaries. PLoS One. 2015;10: e0127616 10.1371/journal.pone.0127616 26061036 PMC4464656

[54] Kelaher BP, Coleman MA, Broad A, Rees MJ, Jordan A, Davis AR. Changes in fish assemblages following the establishment of a network of no-take marine reserves and partially-protected areas. PLoS One. 2014;9: e85825 10.1371/journal.pone.0085825 24454934 PMC3893262

[55] Harasti D, Williams J, Mitchell E, Lindfield S, Jordan A. Increase in relative abundance and size of snapper Chrysophrys auratus within partially-protected and no-take areas in a temperate marine protected area. Frontiers in Marine Science. 2018;5: 208.

[56] Malcolm HA, Williams J, Schultz AL, Neilson J, Johnstone N, Knott NA, et al Targeted fishes are larger and more abundant in “no-take” areas in a subtropical marine park. Estuar Coast Shelf Sci. 2018;212: 118–127.

[57] Nimbs MJ, Larkin M, Davis TR, Harasti D, Willan RC, Smith SDA. Southern range extensions for twelve heterobranch sea slugs (Gastropoda: Heterobranchia) on the eastern coast of Australia. Mar Biodivers Rec. 2016;9: 27.

[58] Harasti D. Range extension and first occurrence of the thorny seahorse Hippocampus histrix in New South Wales, Australia. Mar Biodivers Rec. 2015;8 10.1017/S1755267215000263

[59] Booth DJ, Figueira WF, Gregson MA, Brown L, Beretta G. Occurrence of tropical fishes in temperate southeastern Australia: Role of the East Australian Current. Estuar Coast Shelf Sci. 2007;72: 102–114.

[60] Figueira WF, Booth DJ. Increasing ocean temperatures allow tropical fishes to survive overwinter in temperate waters. Glob Chang Biol. 2010;16: 506–516.

[61] Davies P, Ingleton T, Jordan A, Barrett N. Mapping shelf rocky reef habitats in the Hunter Commonwealth Marine Reserve. Marine Biodiversity Hub, Office of Environment and Heritage NSW; 2016.

[62] Harasti D, Malcolm H, Gallen C, Coleman MA, Jordan A, Knott NA. Appropriate set times to represent patterns of rocky reef fishes using baited video. J Exp Mar Bio Ecol. 2015;463: 173–180.

[63] Fitzpatrick C, McLean D, Harvey ES. Using artificial illumination to survey nocturnal reef fish. Fish Res. 2013;146: 41–50.

[64] Borcard D, Gillet F, Legendre P. Numerical ecology with R. Springer; 2018.

[65] Oksanen J, Blanchet FG, Kindt R, Legendre P, O’hara RB, Simpson GL, et al vegan: Community ecology package R package version 2.5–2. R Development Core Team R: A language and environment for statistical computing Vienna: R Foundation for Statistical Computing 2018; https://www.worldagroforestry.org/publication/vegan-community-ecology-package-r-package-version-117-8

[66] R Core Team. R: A language and environment for statistical computing. R foundation for statistical computing Vienna, Austria; 2018.

[67] Langlois TJ, Fitzpatrick BR, Fairclough DV, Wakefield CB, Hesp SA, McLean DL, et al Similarities between line fishing and baited stereo-video estimations of length-frequency: Novel application of kernel density estimates. PLoS One. 2012;7: e45973 10.1371/journal.pone.0045973 23209547 PMC3510158

[68] Sekhon J. Multivariate and propensity score matching software with automated balance optimization: The matching package for R. Journal of Statistical Software, Articles. 2011;42: 1–52.

[69] Pearson R, Stevens T. Distinct cross-shelf gradient in mesophotic reef fish assemblages in subtropical eastern Australia. Mar Ecol Prog Ser. 2015;532: 185–196.

[70] Puglise KA, Hinderstein LM, Marr M, Dowgiallo CA, MArtinez FA. Mesophotic coral ecosystem research strategy: International workshop to prioritize research and management needs for mesophotic coral ecosystems [Internet]. Jupiter, Florida: Silver Spring, MD: NOAA National Centers for Coastal Research, NOAA Undersea Research Program. NOAA Technical Memorandum NOS NCCOS 98 and OAR 2; 2009. http://www.mesophotic.org/system/publications/pdfs/000/000/287/original/Puglise-2008a.pdf?1492661452

[71] Bongaerts P, Ridgway T, Sampayo EM, Hoegh-Guldberg O. Assessing the “deep reef refugia” hypothesis: focus on Caribbean reefs. Coral Reefs. 2010;29: 309–327.

[72] Winston MS, Taylor BM, Franklin EC. Intraspecific variability in the life histories of endemic coral-reef fishes between photic and mesophotic depths across the Central Pacific Ocean. Coral Reefs. 2017;36: 663–674.

[73] Underwood AJ, Kingsford MJ, Andrew NL. Patterns in shallow subtidal marine assemblages along the coast of New South Wales. Austral Ecol. 1991;16: 231–249.

[74] Connell SD, Irving AD. Integrating ecology with biogeography using landscape characteristics: a case study of subtidal habitat across continental Australia. J Biogeogr. 2008;35: 1608–1621.

[75] Ridgway KR. Long‐term trend and decadal variability of the southward penetration of the East Australian Current. Geophys Res Lett. 2007; http://onlinelibrary.wiley.com/doi/10.1029/2007GL030393/full

[76] Suthers IM, Young JW, Baird ME, Roughan M, Everett JD, Brassington GB, et al The strengthening East Australian Current, its eddies and biological effects—an introduction and overview. Deep Sea Res Part 2 Top Stud Oceanogr. 2011;58: 538–546.

[77] Hobday AJ, Lough JM. Projected climate change in Australian marine and freshwater environments. Mar Freshwater Res. 2011;62: 1000–1014.

[78] Monk J, Ierodiaconou D, Versace VL, Bellgrove A, Harvey E, Rattray A, et al Habitat suitability for marine fishes using presence-only modelling and multibeam sonar. Mar Ecol Prog Ser. 2010;420: 157–174.

[79] Pittman SJ, Christensen JD, Caldow C, Menza C, Monaco ME. Predictive mapping of fish species richness across shallow-water seascapes in the Caribbean. Ecol Modell. 2007;204: 9–21.

[80] Curley BG, Jordan AR, Figueira WF, Valenzuela VC. A review of the biology and ecology of key fishes targeted by coastal fisheries in south-east Australia: identifying critical knowledge gaps required to improve spatial management. Rev Fish Biol Fish. 2013;23: 435–458.

[81] Dayton PK, Thrush SF, Agardy MT, Hofman RJ. Environmental effects of marine fishing. Aquat Conserv. 1995;5: 205–232.

[82] Methratta ET, Link JS. Ontogenetic variation in habitat associations for four flatfish species in the Gulf of Maine-Georges Bank region. J Fish Biol. 2007;70: 1669–1688.

[83] Frank KT, Petrie B, Leggett WC, Boyce DG. Exploitation drives an ontogenetic-like deepening in marine fish. Proc Natl Acad Sci U S A. 2018;115: 6422–6427. 10.1073/pnas.1802096115 29866836 PMC6016777

[84] Macpherson E, Duarte CM. Bathymetric trends in demersal fish size: Is there a general relationship? Inter Research; 1991; http://digital.csic.es/handle/10261/41625

[85] Harasti D, Lee KA, Gallen C, Hughes JM, Stewart J. Movements, home range and site fidelity of snapper (Chrysophrys auratus) within a temperate marine protected area. PLoS One. 2015;10: e0142454 10.1371/journal.pone.0142454 26544185 PMC4636427

[86] Willis TJ, Parsons DM, Babcock RC. Evidence for long‐term site fidelity of snapper (Pagrus auratus) within a marine reserve. N Z J Mar Freshwater Res. 2001;35: 581–590.

[87] Fowler AJ, Huveneers C, Lloyd MT. Insights into movement behaviour of snapper (Chrysophrys auratus, Sparidae) from a large acoustic array. Mar Freshwater Res. 2017;68: 1438–1453.

[88] Bulman C, Althaus F, He X, Bax NJ, Williams A. Diets and trophic guilds of demersal fishes of the south-eastern Australian shelf. Mar Freshwater Res. 2001;52: 537–548.

[89] Stewart J, Hughes JM. Biological and fishery characteristics of rubberlip morwong Nemadactylus douglasii (Hector, 1875) in eastern Australia. Fish Res. 2009;96: 267–274.

[90] Visconti V, Trip EDL, Griffiths MH, Clements KD. Life-history traits of the leatherjacket Meuschenia scaber, a long-lived monacanthid. J Fish Biol. 2018;92: 470–486. 10.1111/jfb.13529 29431226

[91] Logan JM, Young MA, Harvey ES, Schimel A, Ierodiaconou D. Combining underwater video methods improves effectiveness of demersal fish assemblage surveys across habitats. Mar Ecol Prog Ser. 2017;582: 181–200.

